# Assessing patterns of metazoans in the global ocean using environmental DNA

**DOI:** 10.1098/rsos.240724

**Published:** 2024-08-14

**Authors:** Nathan R. Geraldi, Silvia G. Acinas, Intikhab Alam, Josep M. Gasol, María Luz Fernández-de-Puelles, Caterina R. Giner, Santiago Hernández León, Ramiro Logares, Ramon Massana, Pablo Sánchez, Vladimir Bajic, Takashi Gojobori, Carlos M. Duarte

**Affiliations:** ^1^ Red Sea Research Center, King Abdullah University of Science and Technology, Thuwal, Saudi Arabia; ^2^ Computational Bioscience Research Center, King Abdullah University of Science and Technology, Thuwal, Saudi Arabia; ^3^ Institut de Ciències del Mar, CSIC, Barcelona, Catalunya, Spain; ^4^ Center for Marine Ecosystem Research, Edith Cowan University, Joondalup, Western Australia, Australia; ^5^ Instituto Español de Oceanografía, Centro Oceanográfico de Baleares, Palma de Mallorca 07015, Spain; ^6^ Institute for the Oceans and Fisheries, University of British Columbia, UBC-AERL, Vancouver, Canada; ^7^ Instituto de Oceanografía y Cambio Global, IOCAG, Universidad de Las Palmas de Gran Canaria, Unidad Asociada ULPGC-CSIC, Campus de Taliarte, Telde, Gran Canaria, Canary Islands 35214, Spain

**Keywords:** deep sea, diversity, global abundance, metabarcoding, metagenome, metazoan

## Abstract

Documenting large-scale patterns of animals in the ocean and determining the drivers of these patterns is needed for conservation efforts given the unprecedented rates of change occurring within marine ecosystems. We used existing datasets from two global expeditions, *Tara Oceans* and *Malaspina*, that circumnavigated the oceans and sampled down to 4000 m to assess metazoans from environmental DNA (eDNA) extracted from seawater. We describe patterns of taxonomic richness within metazoan phyla and orders based on metabarcoding and infer the relative abundance of phyla using metagenome datasets, and relate these data to environmental variables. Arthropods had the greatest taxonomic richness of metazoan phyla at the surface, while cnidarians had the greatest richness in pelagic zones. Half of the marine metazoan eDNA from metagenome datasets was from arthropods, followed by cnidarians and nematodes. We found that mean surface temperature and primary productivity were positively related to metazoan taxonomic richness. Our findings concur with existing knowledge that temperature and primary productivity are important drivers of taxonomic richness for specific taxa at the ocean’s surface, but these correlations are less evident in the deep ocean. Massive sequencing of eDNA can improve understanding of animal distributions, particularly for the deep ocean where sampling is challenging.

## 1. Introduction

Anthropogenic impacts, such as climate change and overexploitation, are resulting in continued biodiversity loss on land and in the ocean [1,2]. Yet, we can barely understand the consequences of these changes in the ocean as we are far from achieving a comprehensive understanding of animal distribution and diversity in the marine realm, particularly in the open and deep ocean [3,4]. Enhanced knowledge of the open and deep ocean is important given they form one of the largest habitats in the biosphere [5,6]. Moreover, it is difficult to quantify fragile animals with conventional techniques, in particular, gelatinous organisms that forage, and diet studies suggest to be prevalent in the deep sea [7,8]. An enhanced understanding of marine animal distribution and environmental drivers across the ocean is needed to characterize ecosystem structure, and as a baseline for detecting and understanding change in marine communities as indicated by the Convention on Biological Diversity [9] and the United Nations 2030 Agenda for Sustainable Development [10].

Environmental DNA (eDNA) analyses—based on the detection of marker gene sequences (metabarcode or metagenome sequencing)—can identify a wide array of taxa regardless of life stage, while traditional surveys often only measure animals of specific sizes and life stages. Although different definitions of eDNA exist, it can be defined as DNA extracted from an environmental sample, including intra- and extra-cellular DNA, to attain the most all-inclusive taxonomic data [11]. eDNA-based surveys have emerged as a powerful tool to accelerate the description of diversity in the marine environment by complementing traditional sampling methods because they bypass the need to capture or observe organisms [11–13].

**Figure 1.**
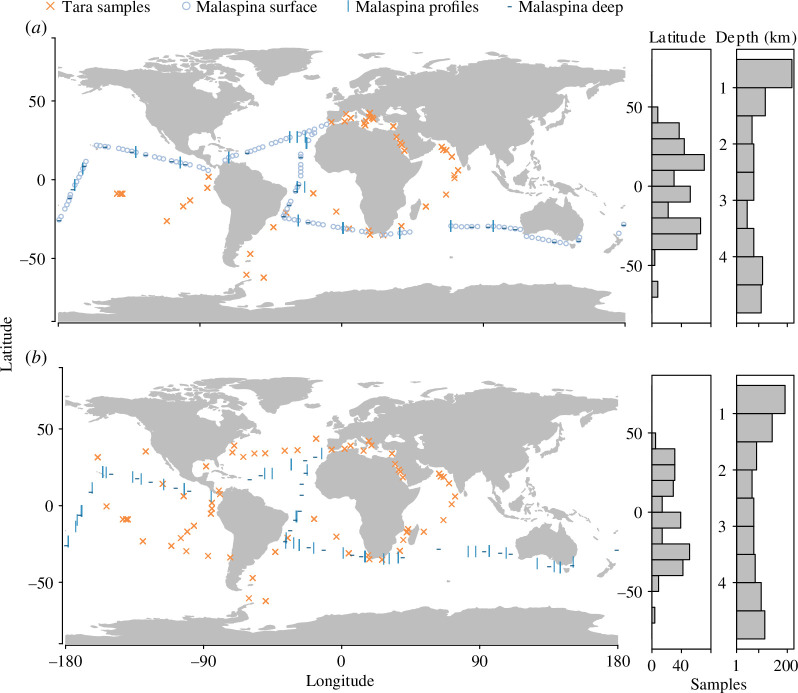
Sample locations for *Tara Oceans* and *Malaspina* global expeditions with sample distribution along the latitude and depth are shown in the right panels for metabarcode (*a*) and metagenome (*b*) datasets.

Here, we utilized metabarcode (PCR amplicons of the 18S rRNA gene) and metagenome (shotgun sequencing of whole community DNA) datasets from already published global expeditions (figure 1). These expeditions targeted microbial size fractions collected from seawater and publications characterized micro-eukaryotes in the photic zone from the *Tara Oceans* expedition [14,15] and in both photic and aphotic zones from the *Malaspina* expedition [16,17]. However, some of these studies reported the presence of metazoan DNA and we aimed to use these datasets to enhance the understanding of the patterns of taxonomic richness and the abundance of marine metazoans in the open and deep ocean based on eDNA. In particular, we used these datasets to (i) describe global patterns of taxonomic richness and the abundance of individual metazoan phyla in the ocean and (ii) identify environmental predictors of taxonomic richness and the abundance of all marine metazoans for the sun-lit and dark ocean.

To determine the predictors of oceanic metazoan richness (from metabarcode datasets) and relative abundance (from metagenome datasets), we chose variables based on the following *a priori* hypotheses. First, taxon richness is expected to increase with temperature but to decrease with depth following multiple theories including metabolic-related hypotheses because temperature is positively related to physiological processes and mutation rates, which may lead to faster speciation rates [18,19], and ‘the more-individuals hypothesis’ based on the positive relationship between the number of individuals and available energy, which also suggests a positive relationship between primary productivity and richness [20,21]. These species richness-energy hypotheses (metabolic theory/kinetic energy) are particularly important for the deep sea [6]. In addition, ‘the more-individuals hypothesis’ inherently suggests a positive relationship between the abundance of taxa and available energy. Second, richness is expected to decrease as the distance to land increases because coastal margins may be a source of diversity to the open and deep ocean [6,22]. Third, greater environmental stability, proxied here as both the annual range in surface temperature and primary productivity, is hypothesized to be positively related to taxonomic richness [23] which could result from stable environments having species with more localized ranges resulting in higher species richness [24]. Finally, human activities can reduce animal abundance and diversity [25] and we used the ocean health index [26], which incorporates harvesting of organisms, global warming and transportation, as a proxy to assess the relationship between anthropogenic impacts and metazoan eDNA. Thus, we assessed the patterns in taxonomic richness and the abundance of marine metazoans from eDNA and the associations of these patterns with environmental and human variables.

## 2. Material and methods

### 2.1. Method overview

This study used metabarcode and metagenome data. These two data types were collected along the survey tracks of both the *Tara Oceans* and the *Malaspina* expeditions (figure 1). Although it is generally accepted that metabarcoding is appropriate for assessing taxonomic richness, biases associated with PCR make metabarcoding’s relationship with abundance less direct and context dependent [27,28]. However, metagenomes utilize shotgun sequencing and may have a better relationship between the abundance of DNA in the environment and post-sequencing data. As a result, we conducted an initial step of comparing results from the two genomic dataset types with morphology-based surveys conducted during the *Malaspina* cruise to determine which genomic data type, if either, relates to abundance and biomass determined by more traditional methods. In light of the results detailed below, we used metabarcode datasets to assess taxonomic richness and metagenome datasets to assess the abundance of marine metazoans. We used these datasets to describe basic patterns in the richness and abundance of the most prevalent metazoan phyla over the ocean’s surface and in the deep ocean, while we used the cumulative taxonomic richness and abundance of all metazoans to analyse relationships with environmental variables. Each expedition had different collection, sequencing and sequence analysis protocols. Thus, we describe and analyse each of the genomic datasets from each expedition separately to make the multiple protocols and findings clear and concise. The expeditions were conducted before eDNA was targeted and thus did not include field or extraction controls that are optimally used to detect contamination and consideration given the lack of these controls in the discussion. However, the expeditions did use common protocols to reduce contamination of samples in the laboratory and details of protocols are given in respective references. For example, PCR controls were always included for each PCR batch and samples were not sequenced if there was amplification in the control. The goal of these expeditions was to maximize the spatial coverage of the collected samples and replicates of samples were not sequenced.

### 2.2. Metabarcoding methods

The *Tara Oceans* sequences are open access and detailed methods are available elsewhere [14,29]. The *Tara Oceans* expedition was conducted from September 2009 to March 2012 and 241 samples from 45 locations were collected, primarily from two or three depths at each location (<400 m depth; figure 1). Sampling occurred day and night and there was no indication that this affected taxonomic richness (supplementary text and electronic supplementary material, figure S1). Before each surface sampling occasion, water was pumped for 10 min before collecting a sample. Before filling containers with samples, they were rinsed with diluted bleach (approx. 1–10%), freshwater twice and collected water twice. After each use, all equipment was rinsed with fresh water. Rinsing equipment with fresh water was sufficient to minimize contamination among samples based on field blanks in a plankton study using similar methods [30]. Water samples were filtered to collect four different size fractions, 0.8–5, 5–20, 20–180 and 180–2000 μm and the filters were flash frozen (other filter size fractions collected during *Tara Oceans* were not used because of inconstant sampling). However, to make results consistent with the other expedition only data from the 0.8–5 µm size fraction were used unless otherwise indicated (see electronic supplementary materials for details). DNA was extracted and a 130 bp section within the V9 region of the 18S rDNA gene (forward/reverse primer-pair 1389F 5′- TTGTACACACCGCCC-3′ and 1510R 5′-CCTTCYGCAGGTTCACCTAC-3′ [31]) was PCR amplified using 5 ng of total DNA template. Illumina compatible adapters with the NEBNext DNASample Prep Master Mix were used to index samples [29]. Sequencing was performed using the Genome Analyser IIx systems (Illumina, San Diego, CA, USA). Sequences were merged using custom fastx software (http://hannonlab.cshl.edu/fastx_toolkit/index.html). Sequences were retained if they had forward and reverse reads and less than 1% error based on the expected error in 50 bp sliding window. Chimeras were removed using the USEARCH program [32]. The V9 sequencing dataset was clustered using the ‘Swarm’ approach [33]. Unique operational taxonomic units (OTU) were removed if they did not occur in at least two samples and had at least three sequences, and taxonomy was assigned using a custom V9_PR2 reference database, primarily containing SILVA data with the ggsearch36 program [14]. These filtering and clustering parameters were used because they were used in analysing these data previously [14,15]. We used clustering methods for all amplicon data because this was the method used previously for this data [14,15], and because OTUs were similar to sequence variants when relating diversity to environmental gradients [34].

The *Malaspina* circumnavigation expedition was conducted from December 2010 to July 2011 (figure 1 [35]), and three separate datasets were collected: surface (119 samples from 119 stations taken at approximately 3 m depth, Logares *et al*. [36]), vertical profiles (86 samples from 13 stations [16]) and deep (26 samples from 26 stations taken at >2000 m [17]). Sampling for *Malaspina* began around 06.00 and finished by 09.00 local time. Niskin bottles were closed at the deepest sample (typically 4000 m depth for deep and profile datasets) and then sequentially shallower samples for the profile dataset. All equipment used during sampling was rinsed several times with MilliQ water and then several times with diluted HCl in between each sample and at the end of the day. Each station within the *Malaspina* vertical profiles usually included seven samples, one from the surface (approx. 3 m), one from the DCM (deep chlorophyll maximum), 2–3 from 200 to 1000 m and 2–3 samples from 1000 to 4000 m. Water was prefiltered through a 200 µm mesh and then sequentially filtered through 20, 3 and 0.2 µm filters (the intermediate filter was 0.8 µm instead of 3 µm for deep samples), which were then flash frozen.

DNA and RNA (RNA only from vertical profile samples) were simultaneously extracted using the Nucleospin RNA kit (Macherey-Nagel) with the NucleoSpin RNA/DNA Buffer Set (Macherey-Nagel) procedures. Samples included the smallest size fraction in surface samples and vertical profiles (0.2–3 µm) and the intermediate size fraction in deep samples (0.8–20 µm). RNA was transcribed to cDNA and the same procedures were followed for both DNA and cDNA. PCR amplification was conducted on a 380 bp section within the V4 region of the 18S rRNA gene (forward/reverse primer-pair TAReukFWD1 5′-CCAGCASCYGCGGTAATTCC-3′ and TAReukREV3 5′-ACTTTCGTTCTTGATYRA-3′ [37]) using 5 ng of the total DNA template. Samples were indexed with Illumina sequencing adapters (i5 and i7) from the Illumina Nextera index kits. Amplicons for the surface and vertical profile samples were sequenced on an Illumina MiSeq platform (2 × 250 bp) and the PCR and sequencing were run at a specialized sequencing company (Research and Testing Lab, USA). Sequences were processed using a custom pipeline [38]. The paired-end reads were merged with PEAR [39], dereplicated and clustered with UPARSE at 99% similarity [40]. The deep samples differed from the other two sample sets and were sequenced on a 454 Life Sciences platform and clustered at 99% similarity using QIIME Uclust [41]. For all *Malaspina* data, taxonomy was assigned using BLAST search on the SILVA 18S database (v. 128 for surface and vertical profiles and 132 for deep samples). Taxonomy assignment to a reference sequence was based on the greatest per cent identity, the max score and then the total score.

Only the sequences (from both expeditions) with taxonomy assigned to Metazoa at a per cent identity match above 90% were retained for analysis. This is greater than past studies using global datasets (80% [14]). Ninety per cent was chosen as a balanced approach to minimize false positives in taxonomic assignment while reducing false negatives by identifying species or taxa not in the reference library, particularly for taxa from the open and deep ocean that are not well represented in reference libraries. All reads were summed for identical taxa (sequences with identical taxonomy) and taxonomic richness was the number of unique taxa for each sample. We present results at higher taxonomic levels (order or higher) given that the Malaspina pipeline did not use a common ancestor algorithm, as well as the rare chance that all three BLAST assignment scores were identical.

### 2.3. Metagenome methods

Samples for the metagenome datasets were collected following similar protocols to the metabarcode datasets and were detailed for *Tara Oceans* [42–44] and the *Malaspina* expeditions [17,45]. There were 200 *Tara Oceans* metagenome samples available in this dataset from 67 locations and 138 unique sampling points, which included three size fraction categories 0.1–0.22, 0.22–3 (including 0.22–0.45, 0.22–1.6, 0.45–0.8 and 0.22–3 μm) and >0.22 μm. These three size categories included 19, 156 and 20 samples, respectively. There were 176 *Malaspina* metagenome samples included in this dataset with deep samples from 31 locations and profile samples from 35 locations and 101 unique location and depth sampling points, which included two size fractions for deep (0.2–0.8 and 0.8–20 μm) and for profile (0.2–3 and 3–20 μm) datasets. Libraries were created and sequenced using Illumina (GAIIx, HiSeq).

All metagenomes were re-annotated, standardized (among samples and calculated as sequences per million), and filtered using the Dragon Metagenomics Analysis Platform (DMAP). Gene abundance estimates from *Tara* samples were used without any modifications. However, for *Malaspina* datasets, we obtained normalized read counts considering the gene length and scaling of sequencing depth to one million reads, represented as fragments per kilobase of transcript per million mapped reads. DMAP assigns taxonomy to sequences from the expeditions using the UniProt database with high-throughput BLASTp using traverse lowest common ancestor from top hits. We searched the metagenome data for single-copy protein-encoding genes, or marker genes, that are taxonomically distinct. We conducted the BLASTp search with three different percent identity matches (PID), 50, 70 and 90. In addition, the search included three separate queries for different marker gene groups to assign sequences to metazoans. Specifically, three different searches of documented [46] enzyme encoding (ec) and mitochondrial domain (pf7) marker genes (search name followed by the exact genes in parentheses) included, ec3 (ec_id:1.6.5.3 OR ec_id:2.7.7.6 OR ec_id:6.3.2.2), ec4 (ec_id:1.6.5.3 OR ec_id:2.7.7.6 OR ec_id:2.7.11.1 OR ec_id:6.3.2.2) and pf7 (hmm_id:PF00189 OR hmm_id:PF01479 OR hmm_id:PF00411 OR hmm_id:PF01248 OR hmm_id:PF00181 OR hmm_id:PF01929 OR hmm_id:PF00252). Thus, nine distinct searches to assign taxonomy to sequences were performed for all metagenome datasets. We chose the query for all analyses (PID and marker gene combination) that had the greatest correlation coefficient (Spearman rank correlation) with morphology-based surveys (see electronic supplementary materials for survey methods). To determine the taxonomic breadth of the reference library, which can indicate biases in the results, the number of sequences for each phylum was obtained for each marker gene query from the UniProt database (https://www.uniprot.org; searched on 8 July 2019). These counts include all sequences as filtering out multiple occurrences of the same species and removing terrestrial species was not feasible with this database.

### 2.4. Statistical analysis

To determine what factors were related to changes in richness and relative abundance of metazoans based on eDNA, we analysed taxonomic richness using metabarcode datasets and abundance using metagenome datasets using either linear models (LM), linear mixed models (LMM) or spatial autoregressive lag and error models (SAR; see electronic supplementary materials for model choice and details). We conducted statistical analyses on the two response variables using eight different datasets: *Tara Oceans* metabarcode and metagenome datasets, *Malaspina* surface metabarcode dataset, *Malaspina* vertical profile DNA and RNA metabarcode datasets, *Malaspina* vertical profile metagenome dataset and *Malaspina* deep metabarcode and metagenome datasets. All analyses were conducted in R, v. 3.4.2 [47]. The datasets from each cruise were analysed separately because they used different methods and targeted different oceanic zones. For metabarcode datasets, the number of reads for each species within each sample was rarefied to the lowest number of reads within a sample for each dataset; 201 505 reads for *Tara* Oceans, 6461 for all *Malaspina* DNA and 21 825 for *Malaspina* vertical profile RNA (rarefy function from vegan package [48]). Rarefaction was chosen because it is a straightforward approach that can be more appropriate than normalization [49]. In addition, to reduce overestimating richness and to be conservative in our calculation of the number of species, reads from different OTUs assigned to the same species were summed and any reads were removed that did not include a species label because it was missing in the matching reference sequence. Finally, the analysis only included samples from similar size fractions (within approx. 0.2–5 µm) to make findings comparable among datasets and reflect findings from morphometric samples.

Predictor variables, chosen *a priori* to be included in statistical models, were sea surface temperature (SST) mean, SST range, surface primary productivity mean, surface primary productivity range, distance to land and the ocean health index. The mean and ranges of SST and primary productivity were extracted from Bio-ORACLE layers that are based on satellite data from 2000 to 2014 [50]. SST and primary productivity ranges were determined by calculating the difference between minimum and maximum temperature for each year of the dataset and then using the mean of these annual ranges. Distance to land was obtained from the global marine environment datasets [51]. The ocean health index is a standardized index of human influence based on many anthropogenic impacts and was used as an overarching proxy for anthropogenic impacts [26]. Details on why we chose surface variables and layer extraction, as well as details about additional analyses on the influence of latitude and the relationship between taxa richness and abundance, are provided in the electronic supplementary material.

## 3. Results

### 3.1. Richness of taxa from metabarcode datasets

**Figure 2.**
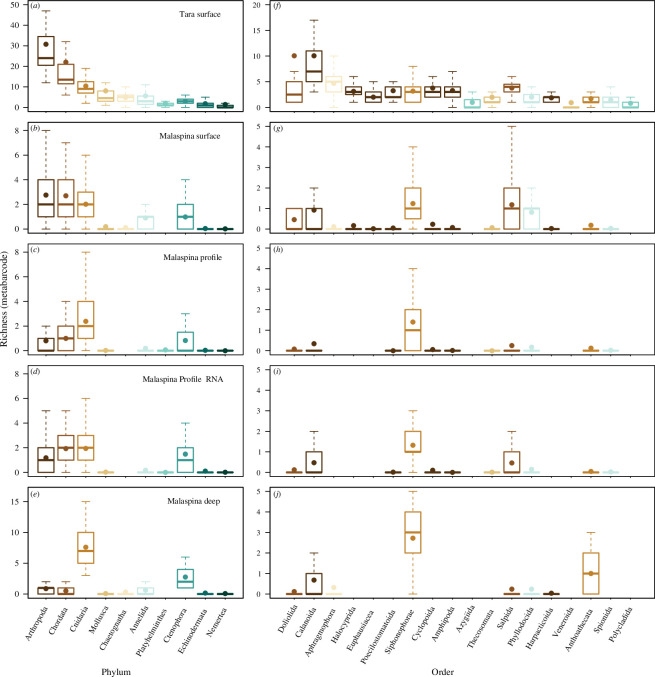
Taxonomic richness of metazoan phyla (*a–e*) and orders (*f–j*) with the greatest taxonomic richness from metabarcode data. Phyla or orders are organized from greatest to least rich (means). Plots show *Tara Ocean* samples (*a* and *f*), *Malaspina* surface samples (*b* and *g*), *Malaspina* profile samples for eDNA (*c* and *h*) and eRNA (*d* and *i*) and *Malaspina* deep samples (*e* and *j*). Only data from comparable filtered size fractions were included (0.2 – 0.8 and 0.2 – 3 μm size fractions for *Malaspina* datasets, and approx. 0.2 – 4.5 μm size fraction for *Tara* Oceans). The colour of the boxes in the second column indicates what phylum the order is in following the left column. Boxplots, based on per sample taxonomic richness, show the median with the upper and lower quartiles, while the whiskers extend to the extreme data point but no more than 1.5 times the respective quartile. The mean is indicated by the circle.

**Figure 3.**
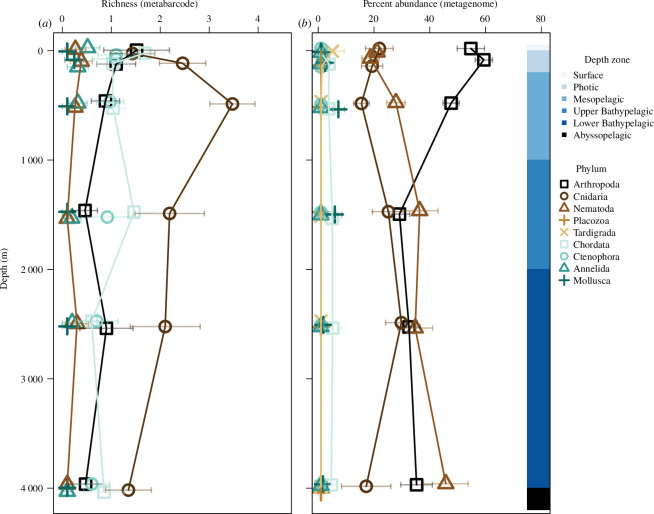
Taxonomic richness (*a*) and per cent abundance (*b*) of sequences within metazoan phyla from the ocean’s surface to the abyssopelagic. Taxonomic richness was from metabarcode data and abundance was from metagenome data, all from the *Malaspina* profile dataset (0.2–3 μm filtered size fractions). Phyla are indicated by shape and colour. The mean taxonomic richness or per cent abundance of samples within the respective categories is shown with symbols along with the standard error indicated by horizontal bars.

Patterns of taxonomic richness within individual metazoan phyla were different at the surface and in the deep ocean. Arthropoda was the phylum with the highest taxonomic richness at the surface of the ocean, especially for the *Tara Oceans* dataset (figure 2*a,b*). The arthropod taxa were primarily from the order Calanoida. Arthropoda included taxa from six additional orders when limited to orders with a mean richness of greater than one per sample (figure 2*f*). Other phyla with high taxonomic richness included Chordata and Cnidaria. The Chordata taxa were primarily from Doliolida and Salpida orders, while the Cnidaria taxon was primarily from Siphonophorae (figure 2*f,g*). In comparison with the *Malaspina* surface data, the *Malaspina* profile data had lower richness within Arthropoda and Chordata, but similar richness of Cnidaria (figure 2*c,h*). *Malaspina* profile eDNA and eRNA had similar patterns of richness for both phyla and orders (figure 2*c,d*,*h*,*i*). The *Malaspina* deep data had greater taxonomic richness within Cnidaria compared with the other metabarcode datasets, which were primarily in Siphonophorae and Anthoathecata orders (figure 2*e,j*). There were changes in the richness of taxa within the major phyla as depth increased within the *Malaspina* profile dataset (figure 3*a*). The richness of arthropod taxon decreased with depth, while there was a peak in the richness of cnidarians within the mesopelagic. *Malaspina* datasets had fewer sequences per sample and a lower percentage assigned to metazoans (0.12% of approximately 97 000 sequences per sample) compared with the *Tara Oceans* (42% of approximately 1.7 million sequences per sample), and this probably resulted in the lower taxonomic richness and why we rarefied and analysed the datasets separately (see electronic supplementary materials for details of sequence depth and assignment). This pattern was counter to the *in silico* results (the supplementary text and electronic supplementary material, figure S2) for which the primers used for Malaspina amplified more metazoans, but the percentage of metazoan sequences is dependent on the amplification of other organisms as well and the primers used for Malaspina probably amplified more microorganisms and at a greater frequency compared with the primers used for *Tara Oceans*.

### 3.2. Relative abundance of taxa from metagenomic datasets

Before we examined the patterns of relative abundance based on eDNA, we needed to determine which genomic protocols to use based on the correlation coefficients between the results of the different protocols and morphological surveys. We found that the most appropriate genomic protocol to use for eDNA-based abundance estimates was metagenomes with a per cent identity matching of >70%, the ec4 marker gene query and only samples from filtered size fractions between 0.2 and 3 μm (electronic supplementary material, figures S3–S6). The sequences identified with this marker gene query and per cent identity match had an *r* ^2^ = 0.73 with morphological abundance and an *r* ^2^ = 0.45 with morphological biomass (electronic supplementary material, figure S5). These relationships were similar to or better than the relationship between abundance and biomass of the morphological surveys (*r* ^2^ = 0.48) and much better than the relationship between metabarcoding sequences and either of the morphological surveys (*r* ^2 ^< 0.11). Although the pf7 query had slightly higher *r* ^2^ values for biomass but not abundance compared with ec4 (electronic supplementary material, figure S3), this query resulted in assigning almost all sequences of the *Tara Oceans* dataset to Mollusca.

The metagenome datasets included 74 species assigned to sequences. This comprised 13 species of arthropods with the majority of sequences assigned to Branchiopoda (>90% of arthropod and 44% of metazoan sequences), 12 cnidarian species with the majority of sequences assigned to Anthazoa (>90% cnidarian and 16% metazoan sequences) and 10 species of nematodes with all sequences assigned to Chromadorea (27% of metazoan sequences).

**Figure 4.**
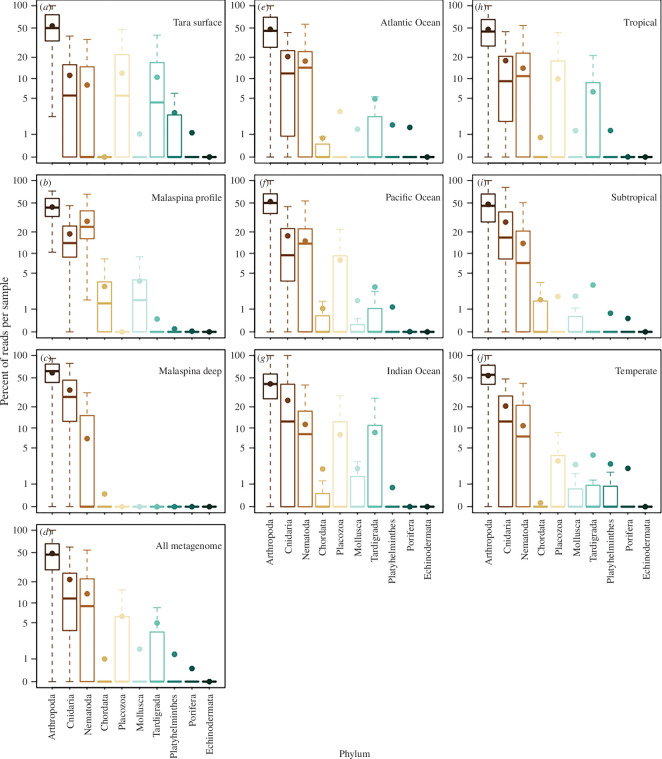
Mean abundance (expressed as the percentage of reads per sample of metazoan marker gene sequences from the metagenomic dataset) of the taxa identified by the two expeditions. Phyla shown are sorted based on highest medians. Plots show *Tara Ocean* samples (*a*), *Malaspina* profile samples (*b*) and *Malaspina* deep samples (*c*), all metagenome samples (*d*), all metagenome data from different ocean basins (*e–g*) and all metagenome data by latitudinal categories (*h–j*). Boxplots, based on per sample data across all samples, show the median with the upper and lower quartiles, while the whiskers extend to the extreme data point but no more than 1.5 times the respective quartile. The mean is indicated by the circle. Only data from comparable filtered size fractions were included (0.2–0.8 and 0.2–3 μm size fractions for *Malaspina* deep and profile datasets respectively and approx. 0.2–4.5 μm size fraction for *Tara Oceans*).

Metagenomes indicated similar median relative abundance patterns among phyla at the surface, for depth profiles, and the deep ocean, as well as for different ocean basins and latitudinal zones, with a few exceptions (figure 4). Arthropods consistently made up 50% of the metazoan eDNA, regardless of dataset or location. Cnidarians were the second most abundant phylum and made up 10–20% of the metazoan sequences. One exception was the *Malaspina* deep samples for which cnidarians made up approximately 30% of the metazoan eDNA. These deep samples were slightly different from the profiles, which indicated that arthropods and cnidarians were more abundant at these depths than nematodes (figure 4*b,c*). Overall, nematodes were the third most abundant phyla and were most abundant in the *Malaspina* profile samples. All other metazoan phyla had eDNA that was undetected in many samples (had medians of zero) with the exceptions of Placozoa and Tardigrada within the *Tara Oceans* dataset, and Chordata and Mollusca within the *Malaspina* profile dataset. At the ocean’s surface, arthropod eDNA (primarily zooplankton) was the most abundant of all metazoan phyla, followed by Cnidaria and Nematoda. However, these three phyla had similar abundances in the bathypelagic zone (figure 3*b*).

### 3.3. Environmental relationships with richness from metabarcode datasets

Overall, we found that temperature and primary production were related to the taxonomic richness of metazoans, however, there were differences among datasets in regard to specific significant predictor variables. The taxonomic richness of all metazoans from the *Tara Oceans* was positively related to the surface primary productivity mean and negatively related to the primary productivity range (electronic supplementary material, figure S7*a* and *b*), but there were also two interactions: one between mean primary productivity and ocean health index and the other between primary productivity range and distance to land (electronic supplementary material, table S1). The interaction between primary productivity and ocean health index was caused by the positive relationship between primary productivity and richness being most evident in areas of greater human impact. The negative relationship between primary productivity range and richness primarily occurred far from the land. The taxonomic richness of metazoans from the *Malaspina* surface dataset was also negatively related to the primary productivity range (electronic supplementary material, figure S7*e*), and positively related to the SST mean and range (electronic supplementary material, figure S7*c,d*). The *Malaspina* profile dataset indicated a positive relationship between richness and primary productivity for both eDNA and eRNA (electronic supplementary material, figure S7*f*, *g*), while eRNA also indicated a negative relationship between richness and primary productivity range (electronic supplementary material, figure S7*h*). There were no significant relationships between taxonomic richness and predictor variables for the *Malaspina* deep dataset (electronic supplementary material, table S1), which included the auxiliary model that included the additional variables, *in situ* temperature and POC flux, at 2000 m. There were no significant relationships between the taxonomic richness and distance to land or ocean health index for any of the datasets. The explanatory power of these models differed among datasets (electronic supplementary material, table S1). The *Tara Oceans*, *Malaspina* surface, and *Malaspina* deep had *r* ^2^ values of 0.59, 0.47 and 0.44, respectively, while the *Malaspina* profile datasets had a low *r* ^2^ value (<0.15).

### 3.4. Environmental relationships with eDNA relative abundance from metagenome datasets

The relative abundance of metazoan eDNA within metagenome datasets was negatively related to depth and SST mean for the *Tara Oceans* metagenome dataset (electronic supplementary material, table S1 and figure S7*i,j*). These same patterns were evident in the *Malaspina* deep dataset (*p*‐value < 0.05), but the Malaspina profile dataset had no significant relationships between eDNA relative abundance and predictor variables. The explanatory power of the models relating metazoan abundance to environmental variables was the highest for the *Malaspina* deep dataset (*r* ^2^ = 0.75), followed by the *Tara Oceans* (*r* ^2^ = 0.36) and *Malaspina* deep datasets (*r* ^2^ = 0.16; electronic supplementary material, table S1). Finally, we found no relationship between the richness and relative abundance of eDNA for any of the datasets with both metabarcode and metagenome data (electronic supplementary material, figure S8).

## 4. Discussion

### 4.1. Global patterns of richness for metazoan phyla eDNA

Given the global coverage of these two expeditions, their combined information probably constitutes the most comprehensive dataset of metazoan eDNA retrieved from the open and deep ocean. These data offered a unique opportunity to enhance our understanding of the distribution and drivers of metazoans throughout the oceans based on eDNA. Patterns at the surface of the ocean indicate that Arthropoda was the most taxonomically rich phylum followed by Chordata and Cnidaria. In addition, the taxonomic richness of some gelatinous phyla increased with depth, including cnidarians, chordates and ctenophores. These basic patterns in the taxonomic richness of metazoan phyla enhance our knowledge of marine diversity from the ocean’s surface to the lesser-studied deep ocean.

Biodiversity and abundance are patchy over the surface of the Earth, particularly, in the oceans, and determining the drivers of such patterns is an objective of ecology because it underpins our ability to understand and mitigate anthropogenic impacts on ecosystems [25]. Multiple theories of biodiversity patterns focus on greater species richness being related to higher temperature and energy [20,21,52]. These hypotheses were supported by broad taxonomic assessments of global taxonomic richness, with temperature being the only variable that was positively related to species richness across multiple taxonomic groups [25,53] and a primary driver for taxonomic richness of copepods identified within *Tara Oceans* metabarcode data [15]. However, the deep ocean may be an exception [53].

This assessment of metazoan taxonomic richness based on metabarcoding indicated that yearly mean SST and primary productivity were positively related to the taxonomic richness of metazoans, while greater ranges of primary productivity were negatively related to richness for the surface of the ocean (electronic supplementary material, table S1 and figure S7). The consistent findings for surface and profile samples from both expeditions, which had different sampling and processing methods, give additional credence to our findings on oceanic patterns—particularly for the *Malaspina* dataset which had limited metazoan reads and low richness per sample (a mean of approx. 10 species per sample). In addition, the relationship between taxonomic richness and environmental variables was consistent between metabarcoding based on DNA and RNA. Although the relationship between eDNA and eRNA is complex, eRNA can be a more direct indicator of the presence of organisms, given that eRNA degrades quicker than eDNA in the environment [16,54]. These findings agree with past global assessments in that mean temperature is a primary driver of species richness [25,53] and that primary productivity is related to the diversity of copepods based on metabarcode data [15]. It is important to consider that our results are based on sampling locations at a single time point, which could influence our findings for areas with high ranges in the predictor variables, as this could also relate to changes in taxonomic richness.

Knowledge of the drivers of global metazoan taxonomic richness in the open ocean, from the surface to the deep sea, is minimal and resource pulses can both increase and decrease diversity [55]. In addition, a study on ophiuroids (brittle stars) throughout the oceans found that deep-sea species richness was positively related to high carbon export from surface primary production and proximity to continental margins, and negatively related to variation in surface primary productivity [6]. Although we found similar relationships with oceanic profiles, we found no support for a relationship between surface primary production and the taxonomic richness of metazoans in the deep ocean. Overall, our findings agree with global assessments that species richness and temperature are positively correlated at the surface of the ocean [25] and that temperature and primary production are positively related to taxonomic richness when samples are taken throughout the water column [6].

We also assessed the influence of latitude (electronic supplementary materials), along with the environmental variables already discussed, on metazoan taxonomic richness. Based on path analysis, SST mean had the strongest causal linkage with richness followed by latitude, and these variables also had a strong causal linkage, indicating that although these variables are correlated, richness is primarily driven by SST and then secondarily by latitude (electronic supplementary material, figure S8). In addition, there were no clear latitudinal trends for either taxonomic richness or the abundance of marine metazoans (electronic supplementary material S9), which is contrary to existing findings using disparate observations through time [53] and to *Tara Oceans* data showing peaks of copepod richness at low latitudes [15]. Perhaps these differences could be a result of single samples versus a culmination of many samples through time and that patterns in specific taxa may not persist in metazoans overall.

### 4.2. Global patterns of metagenomic sequence abundance

Quantification of the global abundance and biomass of organisms is needed for understanding large-scale bio-geochemical cycles and changes in ecosystem structure resulting from anthropogenic impacts. Therefore, finding novel methods to assess abundance can improve our estimates by complementing traditional survey techniques. With regard to eDNA, many studies have found a relationship between the abundance and biomass of organisms and the number of sequences, but there are multiple variables that may alter this relationship [27,56,57]. We found a significant relationship between the relative abundance of marker gene sequences within metagenome datasets and the abundance and biomass measured by morphological methods within metazoan phyla that were identified in both techniques (figure 2*a,b*). We did not find a relationship between biomass and metabarcoding data, which is different from some past studies [14,58], although this relationship is probably taxa dependent [59].

Our findings from metagenome datasets indicate that arthropods, largely represented by crustacean zooplankton, composed approximately 50% of the eDNA, followed by Cnidaria (approx. 11%) and Nematoda (approx. 8%), based on the median per cent of eDNA from marker gene sequences (figure 4*d*). A global estimate of ocean biomass found that arthropods (1.0 Gigatons of Carbon (Gt C)) and fish (0.7 Gt C) had the greatest biomass, followed by gelatinous cnidarians, mollusks and nematodes (0.04, 0.02 and 0.01 Gt C, respectively; [60]). The rank of these biomass estimates compares well with our relative sequence abundance, with the exception of more nematodes and fewer fish. The abundance of chordate DNA (mean of <1%) was orders of magnitude lower than would be expected based on the global biomass estimate (assuming fish biomass to be approximately 70% of arthropod biomass, which would equate to fish being 35% of all metazoan reads). The low relative abundance of fish eDNA is surprising given that the mesopelagic fish biomass may be orders of magnitude greater than previously thought [61]. However, only 0.3% of the DNA sequences were assigned to bony fish. This was unlikely a result of few reference sequences, given that more than one-third of the chordate sequences within the reference database were fish. Another plausible explanation for this discrepancy could be that fish release less eDNA compared with other taxa such as jellyfish [62], resulting in an underestimation of fish abundance based on eDNA. A study using metabarcoding of a section of the CO1 gene also found a low detection of oceanic fishes [30]. An alternative explanation is that the biomass of other phyla is greater in comparison with the estimated fish biomass given the disproportionate sampling of the continental shelf compared with open and deep ocean surveys [5], and the difficulty in sampling soft-bodied and cryptic taxa. In addition, nematode eDNA was prevalent in metagenomes and this is discussed in the electronic supplementary material. Finally, our eDNA-based study suggests that cnidarians had approximately one-fourth of the eDNA of arthropods (based on medians; figure 3*d*), which would suggest that the global abundance of cnidarians may be greater than current existing estimates. These findings offer the first assessment of the relative abundance of animal phyla in the open ocean throughout the globe, based on a single assessment method.

Our ability to measure the abundance of gelatinous taxa may also be enhanced through genomic-based surveys. Delicate gelatinous organisms are notoriously difficult to sample, and the evidence that they have a major role in deep-sea food webs has been, thus far, derived indirectly from stomach content studies [7] or anecdotal observations from submersibles and cameras [8] in specific locations. Our results indicate that the abundance of cnidarian eDNA peaked in the bathypelagic zone (figure 3*b*). The greater abundance of gelatinous cnidarians in the deep ocean (bathypelagic zone) was also evident in the *Malaspina* deep samples, in which cnidarians made up almost one-third of metazoan sequences (figure 3*b–c*). This suggests that cnidarians are particularly prevalent in the deep sea, consistent with inferences derived from indirect sampling [7,8]. Past observations and findings from this study indicate that these gelatinous animals may be more abundant than previously thought in the open ocean and particularly in the deep sea.

The abundance of animals within metazoan phyla may also relate to environmental variables. This follows the ‘the more-individuals hypothesis’ which suggests that more energy (temperature or resources) will lead to more individuals, which in turn will result in more species [21], suggesting that taxonomic richness and abundance may have similar drivers. The abundance of metazoan eDNA was negatively related to depth and mean SST for the *Tara Oceans* samples and a similar trend was found for *Malaspina* deep samples (electronic supplementary material, table S1 and figure S7). Overall, these results agree with current assumptions that the biomass of animals decreases with depth but disagree because abundance did not increase with temperature [20,21]. In addition, comparing the richness and abundance of simultaneously sampled metabarcode and metagenome data indicates little or slightly negative relationship between abundance and taxonomic richness (electronic supplementary material, figure S8). The negative relationship between temperature and eDNA may result from greater preservation of eDNA in colder water [63], which could also affect the relationship between eDNA taxonomic richness and abundance.

### 4.3. Strengths and potential limitations of these datasets and eDNA surveys

Although using a single method enhances comparability among samples compared with using multiple methods, all sampling methods have imperfect detection [57] and a better understanding of biases when using eDNA to survey metazoans is needed. Results from eDNA studies are not based on counting or weighing individuals but on the DNA left from individuals and offer a ‘DNA view’ of the ocean. Sources of eDNA are being investigated and insights from different filtered size fractions can help elucidate eDNA origins (see electronic supplementary materials for a discussion on filtered size fractions).

There are multiple considerations when interpreting eDNA-based datasets. First, results from eDNA studies are limited by the coverage of the reference library used to assign DNA sequences to taxa. The metabarcode reference library did contain many taxa within almost all the major metazoan phyla with the exception of Nemertea, Gastrotricha and Ctenophora and the metagenome database did not have thaliaceans (salps and doliolidas; electronic supplementary material, figure S2 and supplementary text for a detailed discussion on considerations). Improvements in reference databases will increase the diversity detected by eDNA-based surveys. A second consideration with eDNA-based studies is removing false positives while minimizing false negatives. This is particularly important for this study because these datasets were collected before field and extraction controls were common in molecular studies, although PCR controls were run for all datasets and samples were only sequenced if there was no indication of amplification in these controls (see electronic supplementary materials for a detailed discussion on considerations and potential biases of eDNA-based surveys). It is possible that some of the taxa identified and used in our analysis were not present in samples and resulted from contamination. However, these taxa were probably a very small percentage of the total taxonomic richness and we chose our analysis to focus on broad taxonomic groups and the relationship between basic metrics (taxonomic richness) and environmental variables so that the effect of potential contamination would not affect our conclusions, which are extremely valuable given the unprecedented breadth of these data. Although we took many steps to minimize potential biases associated with genomic datasets so that taxonomic richness patterns based on eDNA reflected patterns of actual species richness, the findings should be interpreted knowing that they are based on diversity inferred from 18S rRNA sequences.

## 5. Conclusions

Ocean ecosystems are experiencing significant changes because of anthropogenic impacts [1,2], and determining marine taxa occurrence and environmental properties related to diversity and abundance are needed, especially for the deep ocean [3,4]. Surveys based on eDNA offer a complementary tool to traditional techniques to survey metazoans, particularly for species at low densities or with fragile body forms, as well as in locations difficult to sample such as the deep ocean. Taxonomic richness for all metazoans was positively related to mean SST and mean primary productivity, but negatively related to the range of primary productivity. However, the association with mean SST was not evident for profile samples and no relationship with any of the environmental variables was found for the deep ocean. In general, these findings based on eDNA agree with past findings focusing on specific groups of metazoans and offer a promising tool to survey ocean fauna.

## Data Availability

Sequences and data from the Tara Oceans are available at http://taraoceans.sb731 roscoff.fr/EukDiv/, at EBI under the project IDs PRJEB402 and PRJEB6610, and at PANGAEA. Sequences are available for Malaspina deep samples at the European Nucleotide Archive (http://www.ebi.ac.uk/ena/data/view/PRJEB9943), from Malaspina surface samples at the European Nucleotide Archive (http://www.ebi.ac.uk/ena/data/view/PRJEB23913), and for the Malaspina vertical profile samples at the European Nucleotide Archive (http://www.ebi.ac.uk/ena/data/view/PRJEB23771). R code used for analysis and figures are available at github [64] and data used in the R code are available on Zenodo [65]. Supplementary material is available online [66].
